# The correlation between serum total alkaline phosphatase and bone mineral density in young adults

**DOI:** 10.1186/s12891-022-05438-y

**Published:** 2022-05-18

**Authors:** Juntao Shu, Anjun Tan, Yan Li, Hong Huang, Jingjing Yang

**Affiliations:** 1 Department of Neonatology, The Children’s Hospital of Kunming City, The Affiliated Children’s Hospital of Kunming Medical University, Kunming, 650103 Yunnan People’s Republic of China; 2 Department of Geriatrics, The First People’s Hospital of Yunnan Province, The Affiliated Hospital of Kunming University of Science and Technology, Kunming, 650034 Yunnan People’s Republic of China

**Keywords:** Serum total alkaline phosphatase, Bone mineral density, NHANES, Cross-sectional study

## Abstract

**Background:**

Elevated total alkaline phosphatase (T-ALP) levels are usually indicative of enhanced osteoblastic activity and bone conversion status and are thus considered as a key factor needed for fresh bone mineralization and synthesis. To date, there is no consistent conclusion on the association between the serum T-ALP levels and bone mineral density (BMD). Therefore, the present study focused on exploring the association of serum T-ALP with lumbar BMD among young adults.

**Methods:**

The present cross-sectional study included 6,331 subjects included in the National Health and Nutrition Examination Survey (NHANES) during 2011–2016. The participants aged 20–40 years included 3,349 males and 2,982 females. Serum T-ALP was our main variable, lumbar BMD was our outcome variable, and additional variables were the possible impact modifiers. The relations were analysed by the trend study, weighted multiple linear regression models, smooth curve fitting, and stratified analyses.

**Results:**

In a completely corrected multiple regression model, a negative association between serum T-ALP and lumbar BMD was discovered (β = -0.0007, 95% CI: –0.0009– –0.0005, *P* < 0.000001). After converting the continuous variable serum T-ALP into the categorical one, the significant negative association was still observed (*P* < 0.001), and in the subgroup and smooth curve fitting analyses, this negative correlation remained significant, too.

**Conclusions:**

Our study results indicated that serum T-ALP was negatively associated with lumbar BMD among young adults. Serum T-ALP measurement in the near future might become an effective biomarker to diagnose and treat osteoporosis on time.

## Background

Osteoporosis is a progressive bone disease characterized by decreased bone mineral density and mass caused by a plethora of factors. It can gradually lead to increased bone fragility, and thus, the bone becomes more susceptible to fracture and systemic bone diseases [1]. It is a debilitating disease that affects approximately 200 million people worldwide and demands immediate attention [2]. Due to osteoporosis, there is an imbalance in the trabecular microstructure and the bone remodelling rate, leading to reduced bone strength and a series of clinical symptoms such as discomfort, fracture, and deformity [3]. Thus, early detection and diagnosis of osteoporosis are essential for its effective treatment.

Bone mineral density (BMD) is an fundamental predictor of fracture risk, bone strength, and overall bone condition [4]. Quantifying BMD enables the timely characterization and assessment of osteoporosis and the risk of bone fractures, respectively. However, BMD cannot be used as a solitary measure of a comprehensive evaluation of bone strength [5]. Additionally, it is not particularly valuable as a single monitoring tool for initiating therapy response, as changes in the bone density might be moderate or minor. This is especially notable in the first year of interventional therapy, as DEXA scans cannot adequately detect minor BMD changes [5]. Due to these limitations, more tools, such as biomarkers, need to be explored to support the timely management of osteoporosis [6].

Alkaline phosphatase (ALP) is a homodimer protein with phosphorylation properties and exists as many isozymes [7]. ALP changes can be estimated in a variety of diseases, such as liver disease, cholestatic jaundice, arteriosclerosis, cognitive disorders, and even cerebrovascular diseases [8–12]. Total alkaline phosphatase (T-ALP) and bone-specific alkaline phosphatase (B-ALP) are byproducts produced during bone remodelling. They can be measured in urine or serum and can indicate the bone turnover rate [5]. As serum T-ALP levels have been considered a potential biomarker of bone formation and are widely used in routine screening tests [13], several studies have demonstrated that serum T-ALP is a useful indicator for assessing and tracking the presence or absence of osteoporosis [14]. However, there is no consistent conclusion about the association between serum T-ALP and BDM. Therefore, the present cross-sectional study aimed to determine the association of serum T-ALP with lumbar BMD among young adults included in the National Health and Nutrition Examination Survey (NHANES). All our methods are in accordance with the relevant guidelines and regulations [15–17].

## Methods

### Study population

The NHANES is a population-based cross-sectional survey designed to assemble health and nutrition information on the U.S. household population and is collected every two years. The interview segment included socioeconomic, demographic, health-and diet-associated items, while the physical examinations comprised several laboratory tests [15]. This study was conducted using the data collected in a period from 2011–2016, comprising three NHANES cycles. Our study included 6331 young adults aged 20–40 years and excluded 322 patients with absent serum T-ALP levels, 1878 patients with absent lumbar BMD scores, 66 patients with cancer, and 72 patients with liver disease. Inclusion and exclusion criteria refer to previously published articles [18, 19] and take into account factors that have a greater impact on the main variables, such as cancer and hepatic disease. The ethics review board of the National Center for Health Statistics approved all NHANES protocols, and each participant signed written informed consent (information and details on ethical approvals are available at www.cdc.gov/nchs/nhanes/).

### Variables

Serum T-ALP was the exposure variable in this study. From 2011–2016, the DxC800 system or DxC600i system used the kinetic rate approach that measured plasma or serum T-ALP activity by utilizing 2-amino-2-methyl-1-propanol (AMP) buffer. The coefficient of variation (CV) for the serum T-ALP test was 2.4–7.8%. Our outcome measure was lumbar BMD, which was determined based on DEXA tests. The coefficient of variation (CV) for lumbar BMD measurement was ≤ 0.6%. Several categorical variables, such as race/ethnicity, sex, education, smoking, and drinking habits were considered together with continuous covariates that included age, waist circumference, income to poverty ratio, serum uric acid, blood urea nitrogen, total cholesterol, total protein, serum calcium, serum phosphorus and calcium supplementation levels. Detailed information on the abovementioned covariates, serum T-ALP and lumbar BMD can be found in the National Health and Nutrition Examination Survey: Analytic Guidelines (2011–2016), Laboratory Procedure Manual (2011–2016) and Body Composition Procedures Manual (2011–2016) at http://www.cdc.gov/nchs/nhanes/.

### Statistical analysis

NHANES sample weights were calculated for all estimates to enable a better reflection of the overall characteristics [15, 16]. A weighted multivariate logistic regression model was considered for analysing the correlation of serum T-ALP with lumbar BMD. When the differences between the groups were calculated, the weighted chi-square test (χ^2^) test was used to analyse the categorical variables, and continuous variables were determined by using the weighted linear regression model. Multivariate stratified regression analysis was used to conduct subgroup analysis, while the nonlinear association between serum T-ALP level and lumbar BMD was analysed by generalized additive and smooth curve fitting models. The R package (http://www.R-project.org) and EmpowerStats (http://www.empowerstats.com) were utilized to complete all the data analyses while *P* values < 0.05 were considered statistically significant.

## Results

All 6,331 enrolled participants aged 20–40 years were categorized based on serum T-ALP quartiles (Q1:7–50 IU/L; Q2:51–60 IU/L; Q3:61–74 IU/L; and Q4:75–326 IU/L, Table 1). The basic features were significantly different among diverse serum T-ALP quartiles, except for alcohol consumption and calcium supplementation levels. Relative to other subjects, young adults having the highest serum T-ALP quartile were more likely to be male (61.26%); while the participants with the highest serum T-ALP quartile had a greater waist circumference, total protein, total cholesterol, and serum uric acid levels along with considerably decreased lumbar BMD and income poverty ratio.

**Table 1:** Weighted characteristics of the participants based on serum total alkaline phosphatase quartiles

Serum T-ALP (IU/L)	Total	Q1(7–50)	Q2(51–60)	Q3(61–74)	Q4(75–326)	*P* value
Age (years)	29.94 ± 6.03	29.99 ± 6.02	30.10 ± 5.93	29.56 ± 6.09	29.59 ± 6.13	< 0.0210
Sex (%)						< 0.0001
Male	52.95	41.00	53.95	56.58	61.26	
Female	47.05	59.00	46.05	43.42	38.74	
Race/ethnicity (%)						< 0.0001
Non-Hispanic white	35.98	51.59	48.95	46.02	37.75	
Non-Hispanic black	20.31	19.39	18.15	15.30	16.72	
Mexican American	15.64	7.33	10.19	15.46	23.29	
Other race/ethnicity	28.07	21.69	22.70	23.21	22.24	
Level of education (%)						< 0.0001
Less than high school	16.41	9.25	13.54	16.23	21.25	
High school	21.18	17.65	18.70	21.75	24.43	
More than high school	62.41	73.10	67.76	62.02	54.31	
Income to poverty ratio	2.30 ± 1.55	2.74 ± 1.59	2.52 ± 1.57	2.36 ± 1.55	2.16 ± 1.51	< 0.0001
Smoking behavior (%)						0.0018
Every day	11.96	8.91	11.02	12.72	13.41	
Some days	4.22	3.42	4.78	4.70	3.89	
Not at all	9.59	10.27	10.83	10.74	9.61	
Not recorded	74.24	77.40	73.37	71.84	73.09	
Alcohol consumption (%)						0.0641
High alcohol use	7.80	6.46	7.76	7.49	8.87	
None/moderate alcohol use	49.50	50.64	51.84	51.83	47.82	
Not recorded	42.69	42.90	40.40	40.67	43.31	
Waist circumference (cm)	95.28 ± 17.05	90.17 ± 15.73	94.01 ± 15.60	96.52 ± 16.74	101.46 ± 18.00	< 0.0001
Blood urea nitrogen (mg/dL)	11.70 ± 3.87	11.72 ± 3.76	12.07 ± 3.71	11.82 ± 3.69	11.65 ± 4.13	0.0146
Serum uric acid (mg/dL)	5.35 ± 1.37	5.03 ± 1.37	5.30 ± 1.36	5.43 ± 1.30	5.66 ± 1.33	< 0.0001
Total protein (g/dL)	7.24 ± 0.44	7.15 ± 0.43	7.19 ± 0.42	7.24 ± 0.44	7.28 ± 0.42	< 0.0001
Total cholesterol (mg/dL)	183.44 ± 37.34	178.50 ± 35.03	181.60 ± 36.61	182.92 ± 35.53	188.48 ± 39.68	< 0.0001
Serum phosphorus (mg/dL)	3.77 ± 0.57	3.82 ± 0.56	3.75 ± 0.54	3.77 ± 0.59	3.76 ± 0.61	0.0061
Serum calcium (mg/dL)	9.41 ± 0.33	9.37 ± 0.32	9.41 ± 0.31	9.43 ± 0.34	9.43 ± 0.33	< 0.0001
Calcium supplementation (mg/d)	378.82 ± 139.01	376.48 ± 114.52	377.29 ± 165.36	375.86 ± 119.25	381.71 ± 143.27	0.6294
Lumbar BMD (g/cm2)	1.05 ± 0.14	1.07 ± 0.14	1.06 ± 0.15	1.04 ± 0.14	1.01 ± 0.14	< 0.0001

Mean ± SD for continuous variables: the *P* value was calculated by the weighted linear regression model

Percent (%) for categorical variables: the *P* value was calculated by the weighted chi-square test.

*Abbreviation:* *serum T-ALP* serum Total Alkaline Phosphatase, *BMD* Bone Mineral Density

**Fig. 1:**
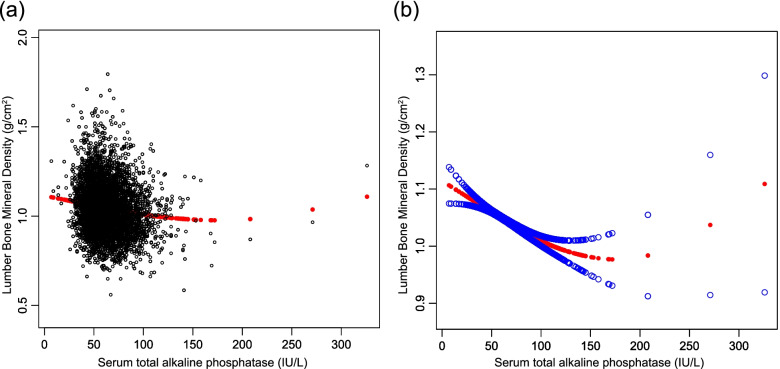
The correlation between serum total alkaline phosphatase and lumbar bone mineral density. **a** Each black point represents a sample. **b** The solid red line represents the smooth fitting curve between variables, and the blue band represents the 95% confidence interval of the fitting. (Age, sex, race/ethnicity, education, income to poverty ratio, waist circumference, smoking behavior, alcohol consumption, blood urea nitrogen, serum uric acid, total protein, total cholesterol, serum phosphorus, serum calcium and calcium supplementation were adjusted)

Table 2 displays a set of multiple regression results. Serum T-ALP showed a negative association with lumbar BMD in the unadjusted model (β = -0.0010, 95% CI: –0.0012– –0.0008, *P* < 0.000001). After adjusting for confounders, this negative association was still present in model 2 (β = -0.0008, 95% CI: –0.0010– –0.0007, *P* < 0.000001) and model 3 (β = -0.0007, 95% CI: –0.0009– –0.0005, *P* < 0.000001). As the serum T-ALP quartile levels were depicted as a categorical variable, the lumbar BMD score gradually decreased with increasing serum T-ALP quartiles which were statistically significant (*P* < 0.001). The BMD of the subjects who were within the greatest quartile decreased by 0.0459 g/cm^2^ as compared to the subjects present within the smallest quartile. Additionally, smooth curve fitting and weighted generalized additive models were applied to assess their association and the conclusions were consistent (Fig. 1).

**Table 2:** The correlation between serum total alkaline phosphatase (IU/L) and lumbar bone mineral density (g/cm2)

	Model 1 β (95% CI) *P* value	Model 2 β (95% CI) *P* value	Model 3 β (95% CI) *P* value
total alkaline phosphatase (IU/L)	-0.0010 (-0.0012, -0.0008) < 0.000001	-0.0008(-0.0010, -0.0007) < 0.000001	-0.0007(-0.0009, -0.0005) < 0.000001
total alkaline phosphatase categories			
Q1 (7–50)	Reference	Reference	Reference
Q2 (51–60)	-0.0136 (-0.0237, -0.0035) 0.008490	-0.0101(-0.0199, -0.0003) 0.042459	-0.0088(-0.0186, 0.0010) 0.077081
Q3 (61–74)	-0.0337(-0.0436, -0.0238) < 0.000001	-0.0263(-0.0360, -0.0167) < 0.000001	-0.0235(-0.0333, -0.0138) 0.000002
Q4 (75–326)	-0.0603(-0.0704, -0.0502) < 0.000001	-0.0519(-0.0618, -0.0420) < 0.000001	-0.0459(-0.0562, -0.0356) < 0.000001
P for trend	< 0.001	< 0.001	< 0.001

Model 1, no covariates were adjusted

Model 2, age, sex, race/ethnicity were adjusted

Model 3, age, sex, race/ethnicity, education, income to poverty ratio, waist circumference, smoking behavior, alcohol consumption, blood urea nitrogen, serum uric acid, total protein, total cholesterol, serum phosphorus, serum calcium and calcium supplementation were adjusted

**Fig. 2:**
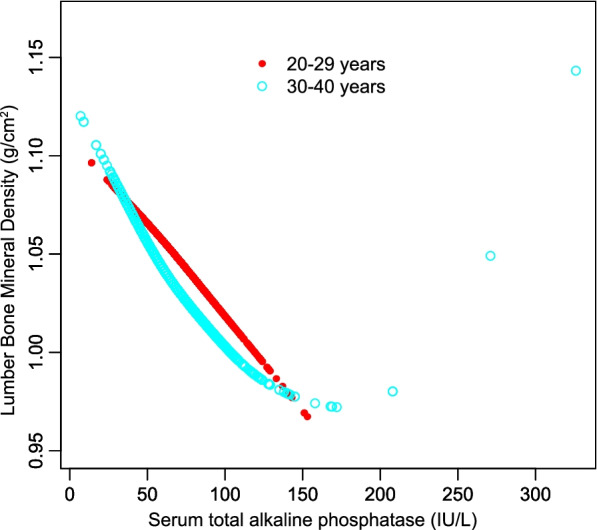
The correlation between serum total alkaline phosphatase and lumbar bone mineral density stratified by age. (Sex, race/ethnicity, education, income to poverty ratio, waist circumference, smoking behavior, alcohol consumption, blood urea nitrogen, serum uric acid, total protein, total cholesterol, serum phosphorus, serum calcium and calcium supplementation were adjusted)

**Fig. 3:**
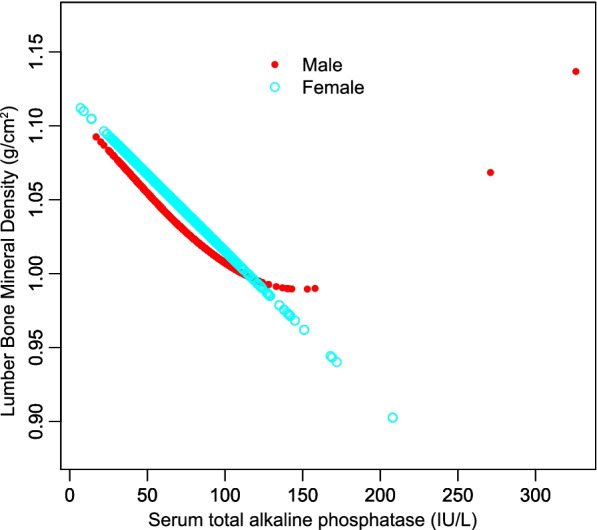
The correlation between serum total alkaline phosphatase and lumbar bone mineral density stratified by sex. (Age, race/ethnicity, education, income to poverty ratio, waist circumference, smoking behavior, alcohol consumption, blood urea nitrogen, serum uric acid, total protein, total cholesterol, serum phosphorus, serum calcium and calcium supplementation were adjusted)

**Fig. 4:**
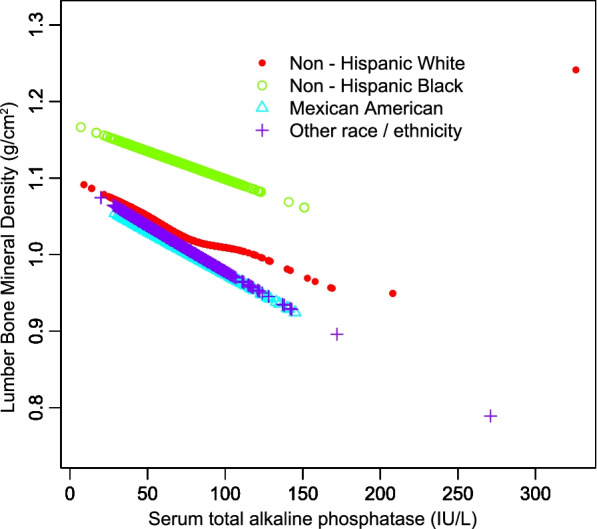
The correlation between serum total alkaline phosphatase and lumbar bone mineral density stratified by race/ethnicity. (Age, sex, education, income to poverty ratio, waist circumference, smoking behavior, alcohol consumption, blood urea nitrogen, serum uric acid, total protein, total cholesterol, serum phosphorus, serum calcium and calcium supplementation were adjusted)

The negative association between serum T-ALP and lumbar BMD remained significant in age, sex, and race-stratified subgroup analyses (Table 3). Furthermore, a smooth curve fitting was executed to assess their nonlinear relationship with each other; thus, leading to consistent results. (Figs. 2, 3, and 4).

**Table 3:** Stratified analysis of the correlation between serum total ALP and lumbar BMD

	Model 1 β (95% CI) *P* value	Model 2 β (95% CI) *P* value	Model 3 β (95% CI) *P* value
Subgroup analysis stratified by age			
20 to 29 years	-0.0013(-0.0016, -0.0010) < 0.000001	-0.0012(-0.0015, -0.0009) < 0.000001	-0.0010(-0.0013, -0.0007) < 0.000001
30 to 40 years	-0.0008(-0.0011, -0.0006) < 0.000001	-0.0006(-0.0008, -0.0004) < 0.000001	-0.0005(-0.0008, -0.0003) 0.000006
Subgroup analysis stratified by sex			
Male	-0.0008(-0.0011, -0.0005) < 0.000001	-0.0007(-0.0009, -0.0004) < 0.000001	-0.0006(-0.0008, -0.0003) 0.000015
Female	-0.0012(-0.0014, -0.0009) < 0.000001	-0.0011(-0.0013, -0.0008) < 0.000001	-0.0010(-0.0012, -0.0007) < 0.000001
Subgroup analysis stratified by race/ethnicity			
Non-Hispanic white	-0.0006(-0.0009, -0.0003) 0.000031	-0.0005(-0.0008, -0.0003) 0.000140	-0.0004(-0.0007, -0.0002) 0.002834
Non-Hispanic black	-0.0009(-0.0013, -0.0004) 0.000187	-0.0010(-0.0014, -0.0005) 0.000059	-0.0007(-0.0012, -0.0003) 0.002721
Mexican American	-0.0012(-0.0015, -0.0008) < 0.000001	-0.0011(-0.0015, -0.0007) < 0.000001	-0.0010(-0.0014, -0.0006) < 0.000001
Other race/ethnicity	-0.0013(-0.0017, -0.0010) < 0.000001	-0.0013(-0.0016, -0.0009) < 0.000001	-0.0013(-0.0017, -0.0010) < 0.000001

Model 1, no covariates were adjusted

Model 2, age, sex, race/ethnicity were adjusted

Model 3, age, sex, race/ethnicity, education, income to poverty ratio, waist circumference, smoking behavior, alcohol consumption, blood urea nitrogen, serum uric acid, total protein, total cholesterol, serum phosphorus, serum calcium and calcium supplementation were adjusted. In the subgroup analysis stratified by age, sex and race/ethnicity, the model was not adjusted for age, sex or race/ethnicity

## Discussion

One of the study findings suggested that participants in the highest serum T-ALP quartile had increased waist circumference, total protein, total cholesterol, and serum uric acid levels as well as lower lumbar BMD and a lower income poverty ratio which was consistent with previously published studies. A retrospective study conducted at King Fahd Hospital, Khobar University, suggested that serum T-ALP levels can be used as a predictor of osteoporosis in combination with BMD in evaluating osteoporosis, while controlling dyslipidaemia levels might improve overall bone health [20]. As another study reported that increased blood uric acid levels played a significant role in osteoporosis [21], these variables were adjusted accordingly in our study.

The relationship of serum T-ALP with BMD score has rarely been investigated before, which was attempted in our study. Based on our multiple regression analysis, serum T-ALP showed a negative association with lumbar BMD score. The depiction of the serum T-ALP quartile levels as categorical variables led to a decrease in the BMD of subjects within the greatest quartile by 0.0459 g/cm^2^ when compared with the subjects within the smallest quartile. Statistical negative significance was observed when the association of serum T-ALP level with lumbar BMD was examined in all sex-, age-, and race-stratified subgroup analyses, while the conclusions from smooth curve fittings were consistent. Elevated serum T-ALP levels are usually indicative of bone formation activity [20]. In our study, the negative correlation between serum T-ALP and lumbar BDM may be explained by the following factors. First, osteoporosis leads to a decline in bone strength, which is determined by bone mass and bone quality [22]. Bone mass is mainly expressed by BMD, while bone quality is determined by microstructure, bone turnover, mineralization and microdamage accumulation [23]. Serum T-ALP is one of the markers reflecting osteogenic activity in bone turnover [5], and it is synthesized during bone matrix maturation and is closely related to bone matrix mineralization [24]. When BMD is low, static osteoblasts are stimulated to become active osteoblasts. This results in bone-like tissue that cannot be mineralized and osteoblasts cannot be transformed into osteocytes. Osteoblasts proliferate in feedback, synthesize large amounts of B-ALP, the serum T-ALP increases significantly. Second, osteogenic activities, including serum T-ALP, are not absolutely independent of osteoclastic activities in the process of bone turnover [25]. In other words, there is some interaction between the two processes, and elevated serum T-ALP may indicate accelerated bone turnover. the negative correlation between bone turnover markers and BMD is more pronounced in the elderly population, suggesting that accelerated bone turnover underlies age-related bone loss [26]. These are possible reasons for the negative correlation between the two. Of course, the underlying mechanism needs to be further examined by in future studies.

A retrospective study of 3242 adults in southern Taiwan showed that serum T-ALP levels were inversely associated with BMD and T-score [27]. It was evident that postmenopausal women demonstrated a more obvious negative correlation between serum T-ALP and osteoporosis [28, 29]. However, our study found that male subjects occupied a large proportion within the greatest serum T-ALP quartile due to age and ethnic differences in the survey population. Our study concluded that higher serum T-ALP levels are associated with decreased lumbar BMD, which is consistent with other reported studies [30–32]. However, an in vitro study used Alizarin Red S staining and serum T-ALP measurements in cultured bone marrow mesenchymal stem cells to evaluate osteogenesis and suggested that increased BMD and T-ALP activity can prevent OVX-induced osteoporosis in rats [33]. Furthermore, a cross-sectional Pakistani study showed no correlation between BMD and serum T-ALP levels by linear regression analysis (*p* = 0.869) [13]. Thus, further studies based on larger statistical samples are required to substantiate the significance of the serum T-ALP and BMD correlations.

### Strengths and limitations

Our study used a weighted nationally representative sample containing a multiracial population to ensure that the results were highly representative of the whole population. A sufficient sample size ensured further subgroup analysis according to the STROBE statement [17]. Nevertheless, several limitations of our research must be acknowledged. First, because of the cross-sectional nature of the study, it was impossible to conclude the causal relationship of serum T-ALP levels with lumbar BMD among young adults. Consequently, more prospective studies with a larger sample size must be initiated to illustrate the underlying mechanism that might link serum T-ALP and BMD. Second, our study was not representative of the population with malignant tumours and liver disease, as individuals with these diseases were excluded to rule out their possible significant influence on lumbar BMD and serum T-ALP levels. Finally, there were additional possible unadjusted confounders that could still lead to bias.

## Conclusions

To conclude, our research indicates a negative association between serum T-ALP levels and lumbar BMD scores in young adults. This relationship remained significant after conducting the subgroup and smooth curve fitting analyses. Subsequently, it is also suggested that serum T-ALP measurement might become an effective biomarker for diagnosing and treating osteoporosis in future cases.

## Data Availability

The datasets generated and/or analysed during the current study are available in the Health and Nutrition Examination Survey (NHANES) during 2011–2016, at http://www.cdc.gov/nchs/nhanes/.

## Abbreviations

T-ALP: Total Alkaline Phosphatase
BMD: Bone mineral density
NHANES: National Health and Nutrition Examination Survey
